# Supplementary material for: RBPMS inhibits bladder cancer metastasis by downregulating MYC pathway through alternative splicing of ANKRD10
Source: Commun Biol. 2025 Mar 5;8:367. doi: 10.1038/s42003-025-07842-1 (PMC11882939; doi:10.1038/s42003-025-07842-1)
Supplement: Supplementary file 2 — Supplementary information [file 42003_2025_7842_MOESM2_ESM.pdf]

Supplementary Information

RBPMS inhibits bladder cancer metastasis by downregulating MYC pathway through alternative splicing of ANKRD10

Supplementary Figures 1-7: Pages 2-13

Supplementary Tables 1-6: Pages 14-38

Supplementary Figures 1-6

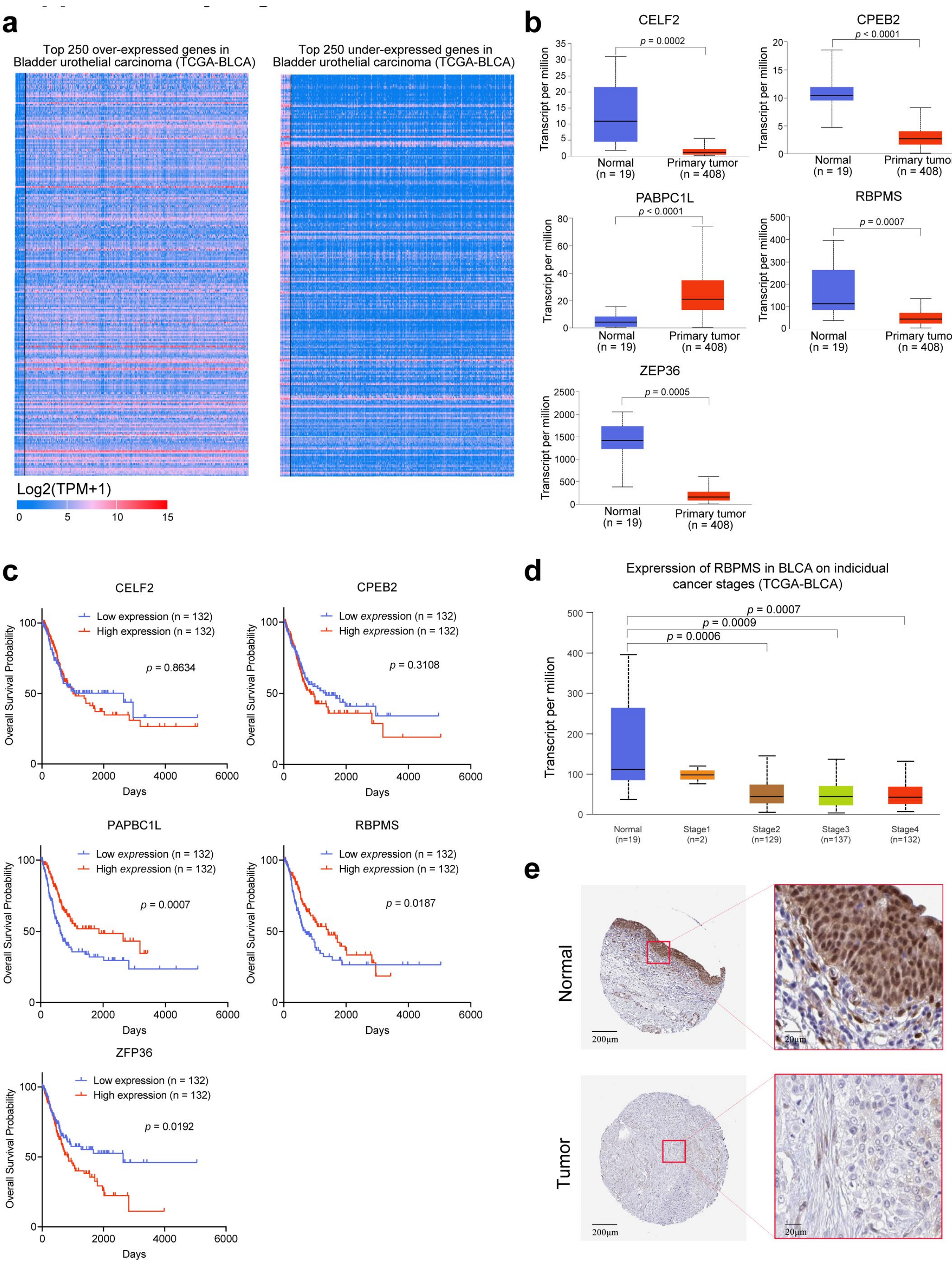

Supplementary Figure 1. RBPMS expression is reduced in BLCA progression and correlated with poor prognosis.

a Heatmap showing the 250 genes with the highest (left) and 250 genes with the lowest (right) fold change in

differential ploidy for cancer and paracancer tissues in TCGA-BLCA cohort. **b** Analysis of mRNA levels of cancer (n = 408) and paracancer tissue (n = 19) expression of RNA binding proteins in TCGA-BLCA cohort. **c** The patients were categorized into a group with high mRNA levels (n = 132) and a group with low mRNA levels (n = 132) based on the median gene expression in the TCGA-BLCA cohort (RNA-seq data). The patients with missing survival data were not included. Statistical significance of survival data was ascertained by the log-rank test of Kaplan–Meier analysis. **d** mRNA levels of *RBPMS* across various stages of bladder cancer and paracancer tissues were evaluated within the TCGA-BLCA cohort. **e** Immunohistochemical staining of RBPMS in normal bladder and bladder cancer tissues from The Human Protein Atlas database. Data are shown as mean ± SD.

Supplementary Figure 2

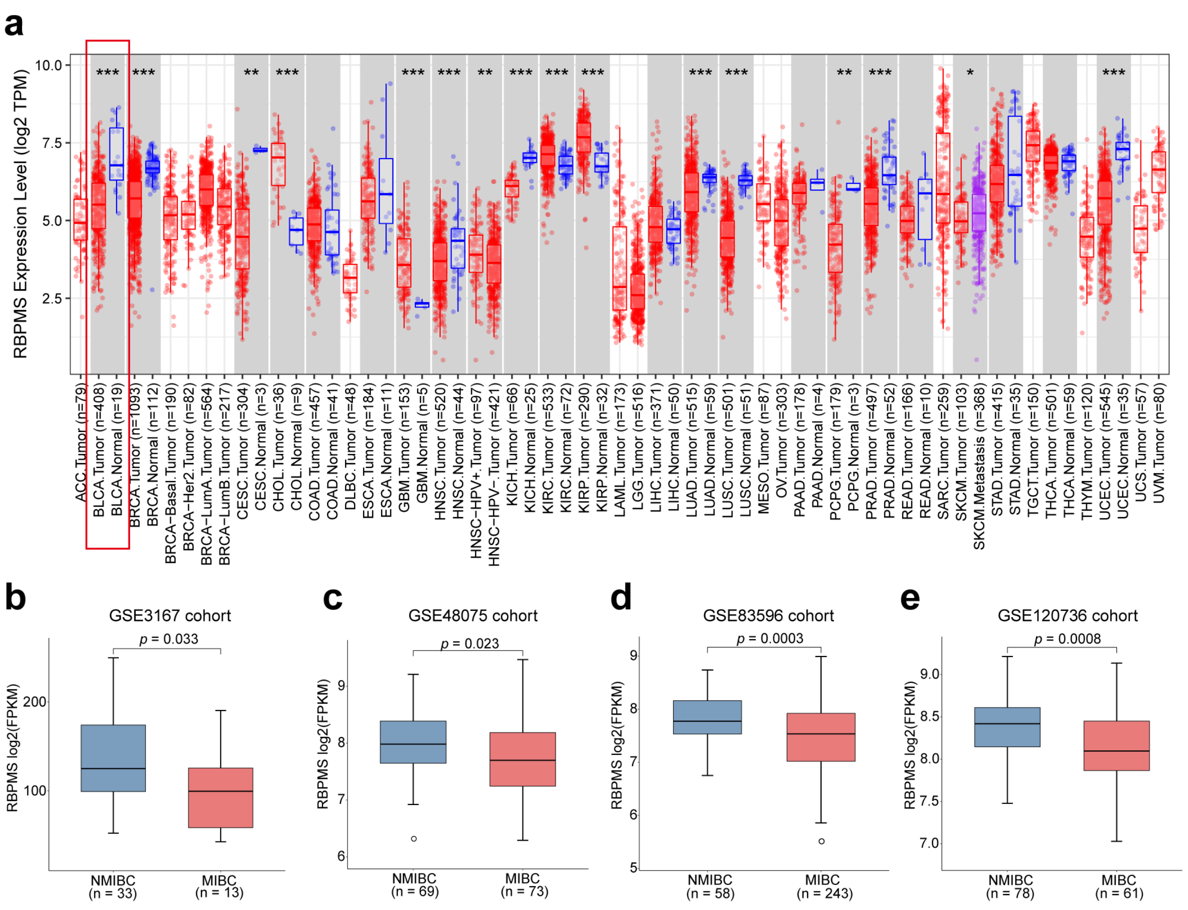

Supplementary Figure 2. Bioinformatics analysis of RBPMS in pan cancer.

a The mRNA expression profiles of *RBPMS* across various cancer tissues and their corresponding adjacent normal tissues were assessed within the TCGA-BLCA cohort. **b-e** mRNA levels of *RBPMS* in MIBC and NMIBC in GSE3167, GSE48075, GSE83596, and GSE120736 cohorts. Data are shown as mean ± SD. *: $p < 0.05$, **: $p < 0.01$, ***: $p < 0.001$.

Supplementary Figure 3

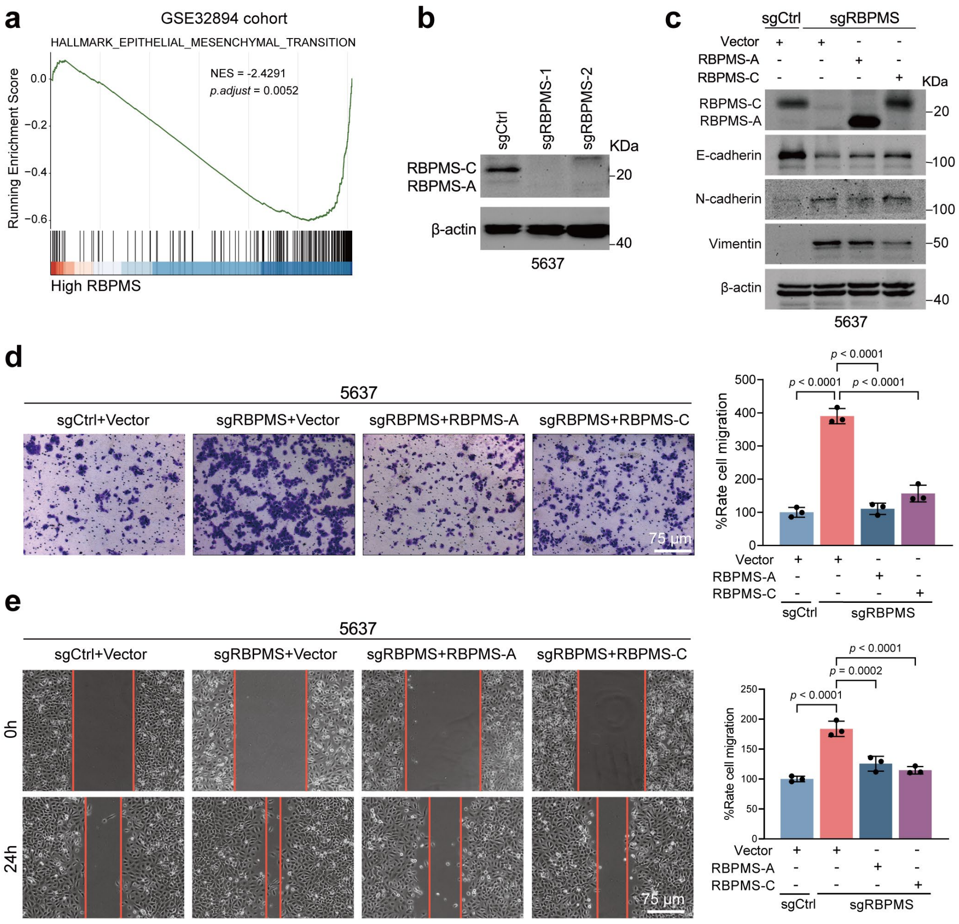

Supplementary Figure 3. Knockout of RBPMS promotes metastasis of bladder cancer.

a GSEA of GSE32894 cohort show RBPMS was negatively related to epithelial mesenchymal transition pathway. **b** CRISPR plasmid-mediated knockout of *RBPMS* was transfected into 5637 cells, followed by the assessment of protein expression levels using Western blot analysis. **c** *RBPMS* was knocked down in 5637 cells before transfection with RBPMS-A or RBPMS-C plasmids. Changes in the levels of EMT pathway-related proteins were determined by Western blot analysis. **d** Representative image (left) and cell number statistical graph (right) of transwell assays from the indicated groups with RBPMS-A or RBPMS-C overexpression in 5637 sgRBPMS cells (n = 3). **e** Representative image (left) and cell number statistical graph (right) of wound healing assays from the indicated groups with RBPMS-A or RBPMS-C overexpression in 5637 sgRBPMS cells (n = 3).

Supplementary Figure 4

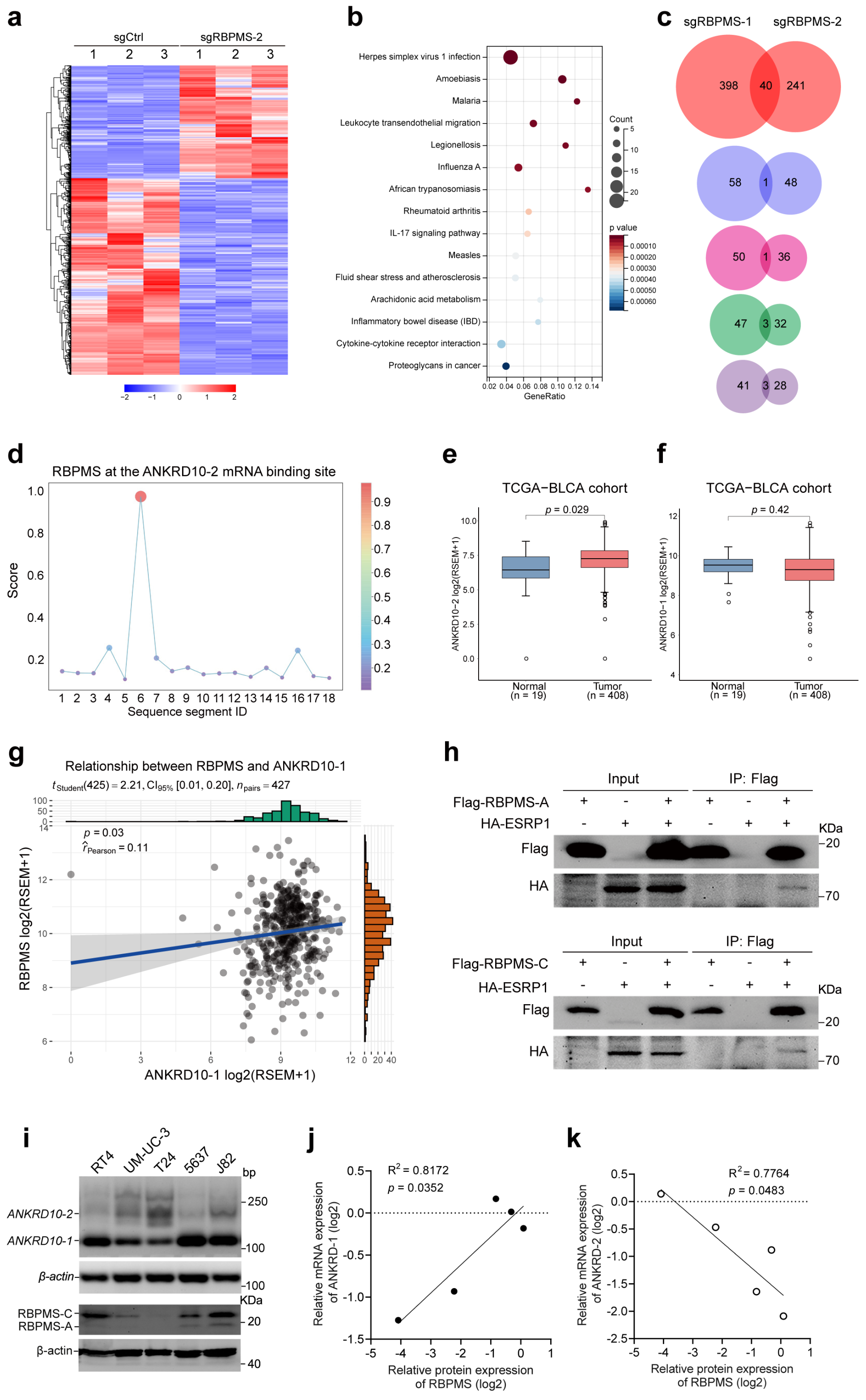

Supplementary Figure 4. RBPMS regulates alternative splicing of ANKRD10.

a Heatmap illustrating the gene expression profiles of the 5637 sgCtrl and sgRBPMS-2 cell lines. **b** KEGG enrichment analysis of DEGs in RNA-seq results after knockout of *RBPMS*. **c** The amounts of different ASEs

in the RNA-seq results are shown by pie charts (p-value < 0.05). **d** Predicted RBPMS binding site scores on *ANKRD-2* mRNA on the RBPsuite website (http://www.csbio.sjtu.edu.cn/bioinf/RBPsuite/). The horizontal coordinate each segment represents a nucleotide fragment of 70 bp size. **e, f** mRNA levels of *ANKRD10-1* (g) and *ANKRD10-2* (h) in BLCA and normal tissues in the TCGA-BLCA cohort. **g** Pearson correlation analysis was conducted to examine the correlation in mRNA expression between *RBPMS* and *ANKRD-1* within the TCGA-BLCA cohort. **h** 293T cells were transfected with the described plasmids for 48 hrs, and co-IP was performed with anti-Flag antibody. Data are shown as mean ± SD. **i** The mRNA expression level of *ANKRD10* and protein expression level of RBPMS in BLCA cell lines were detected using RT-PCR and western blotting, respectively. **j, k** Correlation analysis of RBPMS protein levels and mRNA levels of *ANKRD10-1* (j) or *ANKRD10-2* (k) in BLCA cell lines.

Supplementary Figure 5

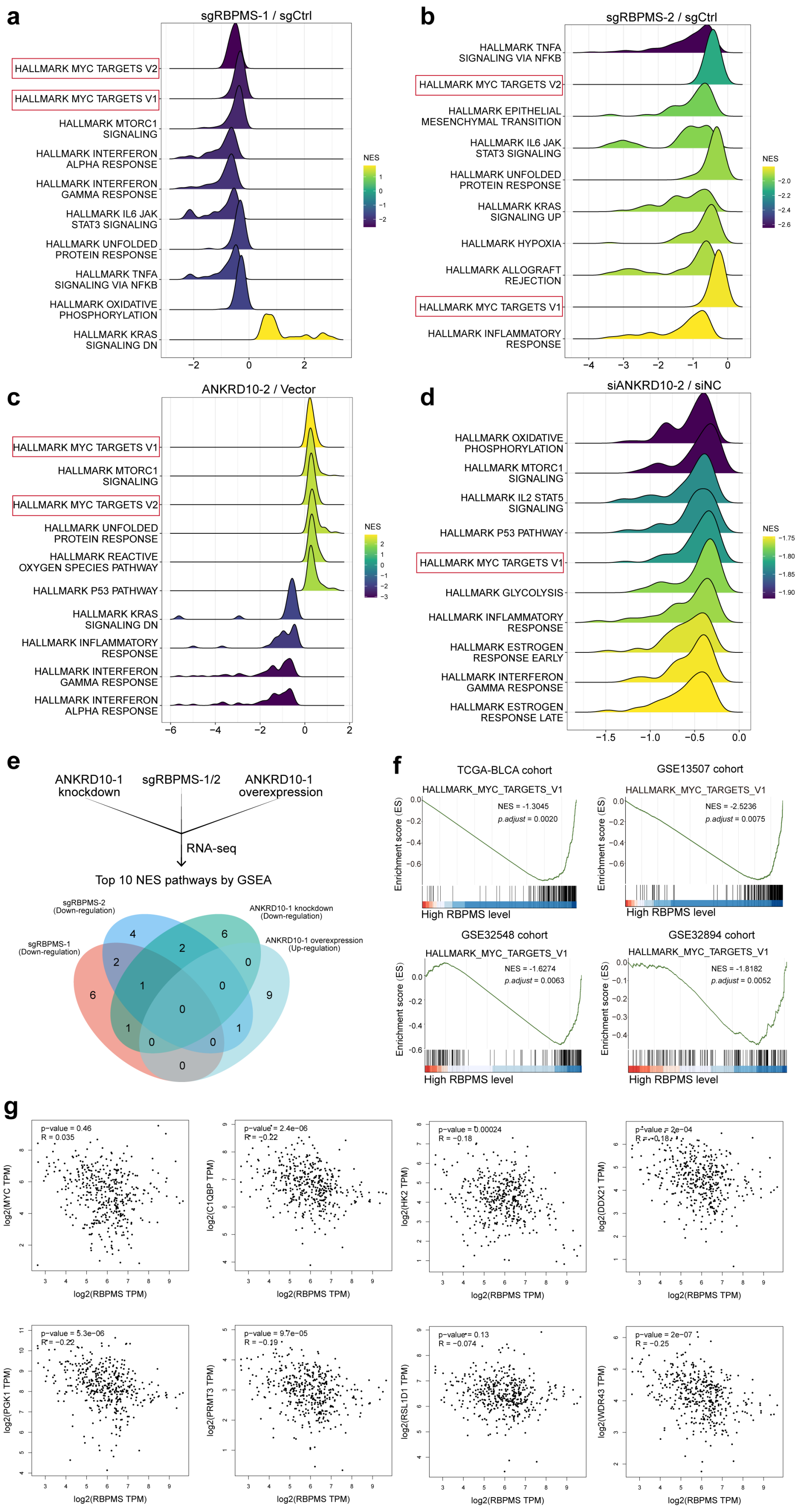

Supplementary Figure 5. RBPMS and ANKRD10-2 are significantly correlated with MYC target gene expression.

a, b Hallmark gene sets (*https://www.gsea-msigdb.org/gsea/msigdb*) related to RBPMS from enrichment analysis of the gene expression matrix from RNA-seq assays. **c, d** Hallmark gene sets related to ANKRD10-2 from enrichment analysis of the gene expression matrix from RNA-seq assays. **e** RNA-seq data were analyzed using GSEA, and pathways down-regulated by knockout of *RBPMS* and knockdown of *ANKRD10-1* were taken to intersect with pathways up-regulated by overexpression of ANKRD10-1. The pathways obtained by GSEA enrichment were selected as the 10 with the smallest p.adjust values. **f** The TCGA-BLCA, GSE13507, GSE32548, and GSE32894 cohorts underwent GSEA analysis to show that MYC target genes were enriched in RBPMS low-expression samples. **g** Pearson correlation analysis of RBPMS with MYC target genes for all bladder cancers and paraneoplastic normal tissues in TCGA and GTEx databases.

Supplementary Figure 6. Original uncropped Western blots.

Figure 2d

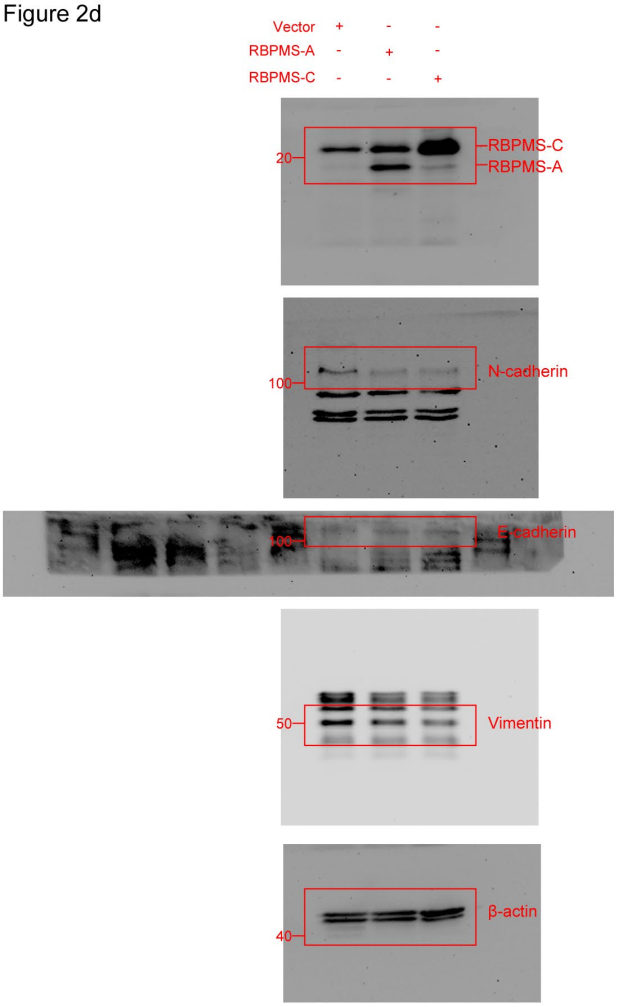

Figure 3j

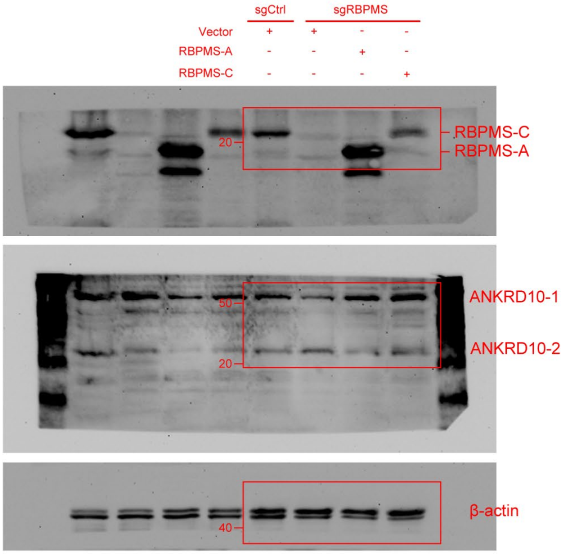

Figure 5h

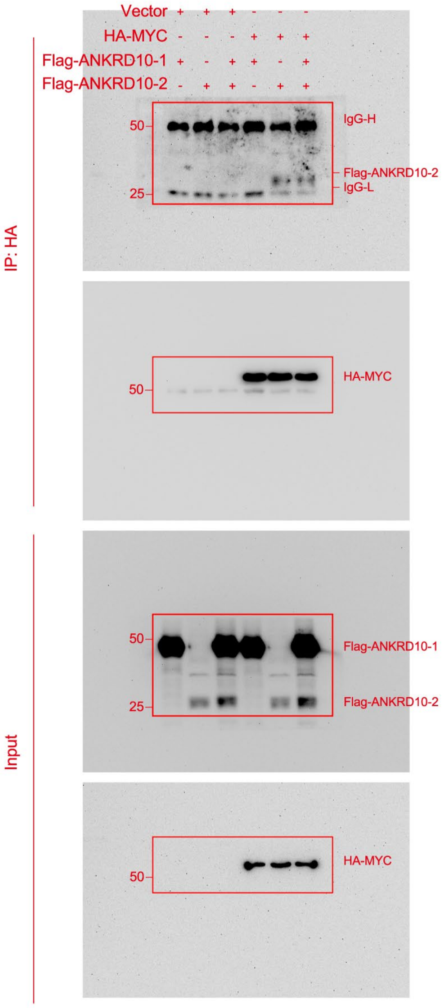

Figure 5g

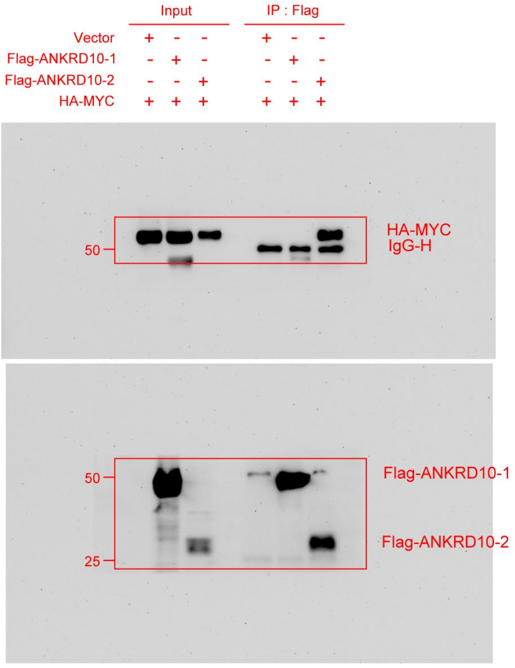

Supplementary Figure 6. Original uncropped Western blots.

Figure S3b

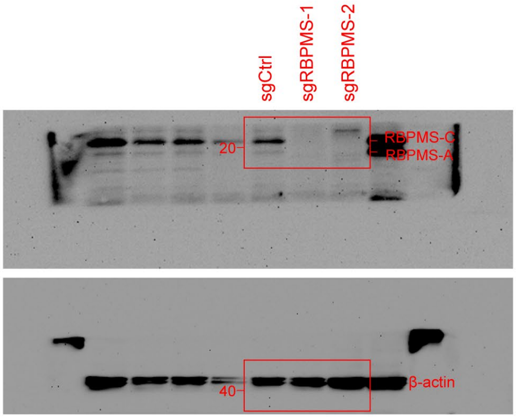

Figure S3c

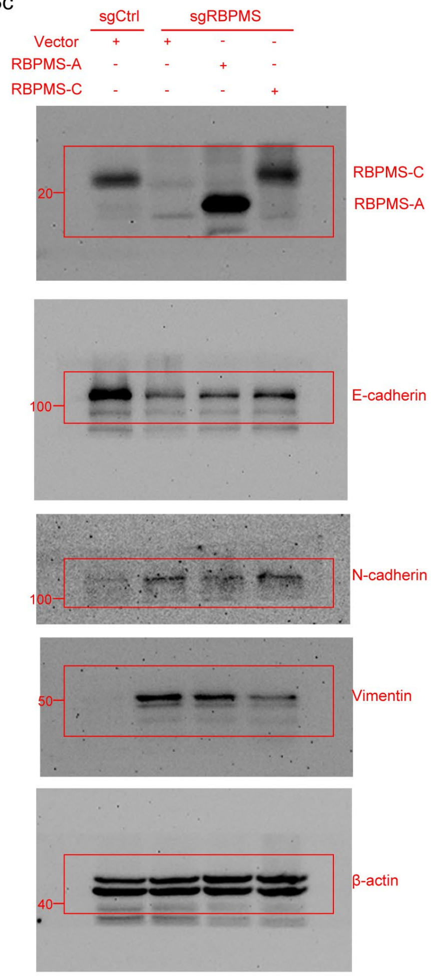

Figure S4h

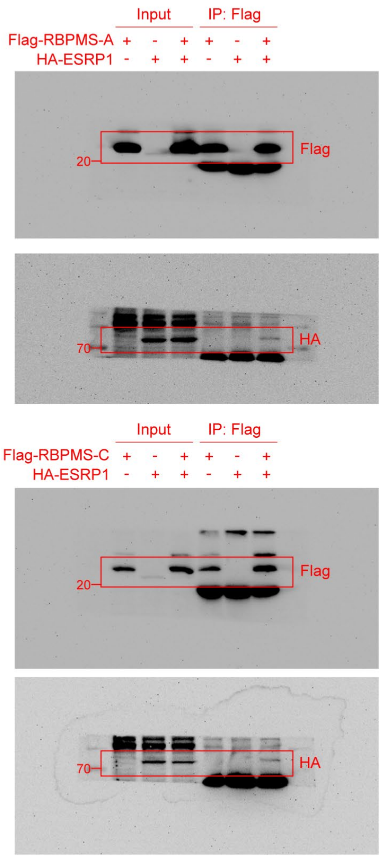

Figure S4i

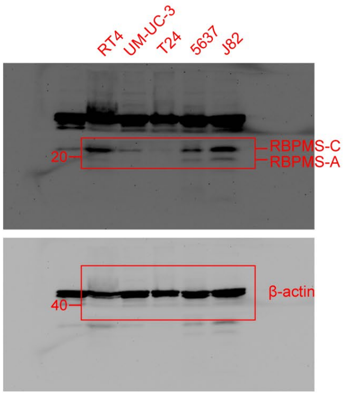

Supplementary Figure 6. Original uncropped Western blots.

Supplementary Figure 7. Original uncropped agarose gels.

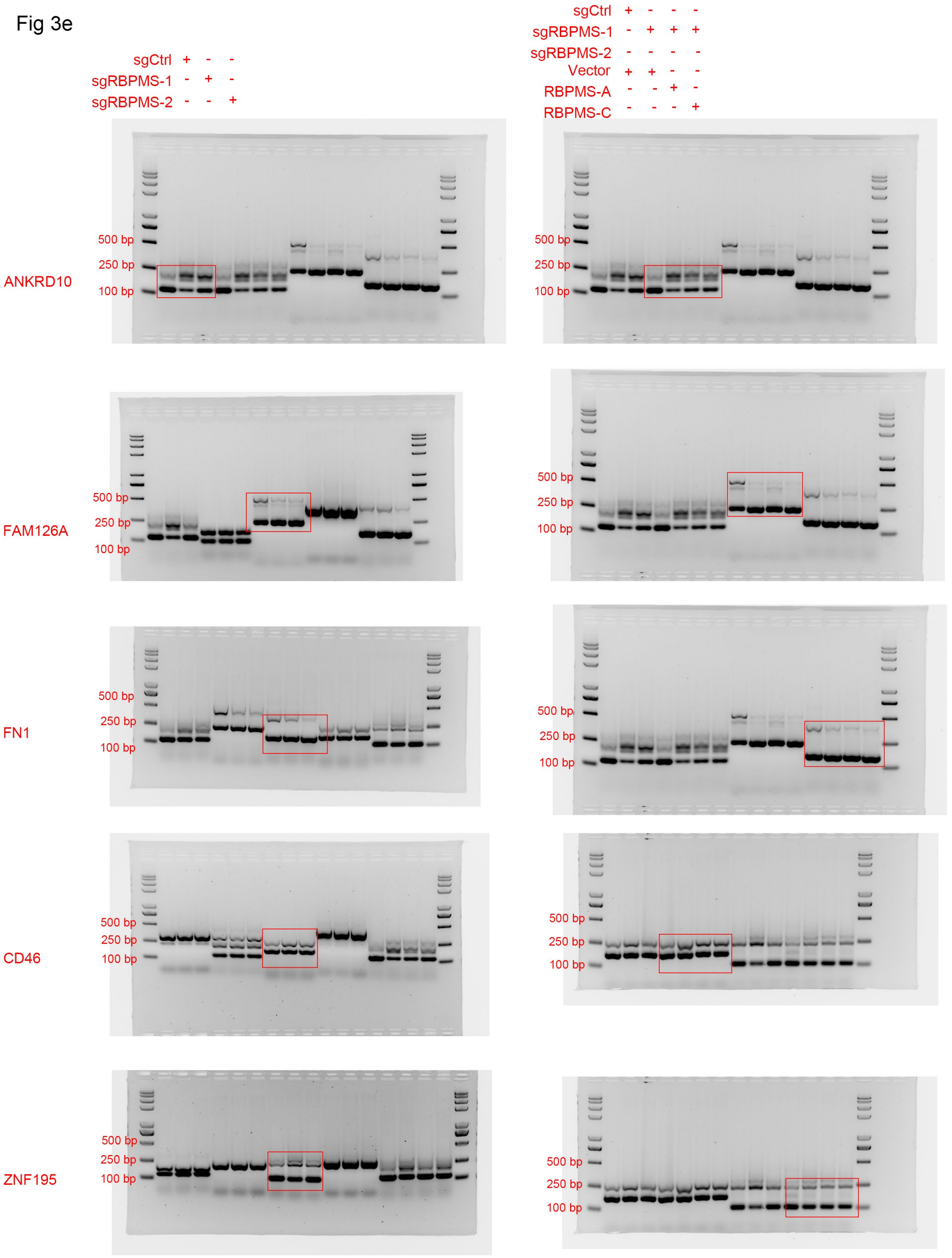

Supplementary Figure 7. Original uncropped agarose gels.

Fig 3e

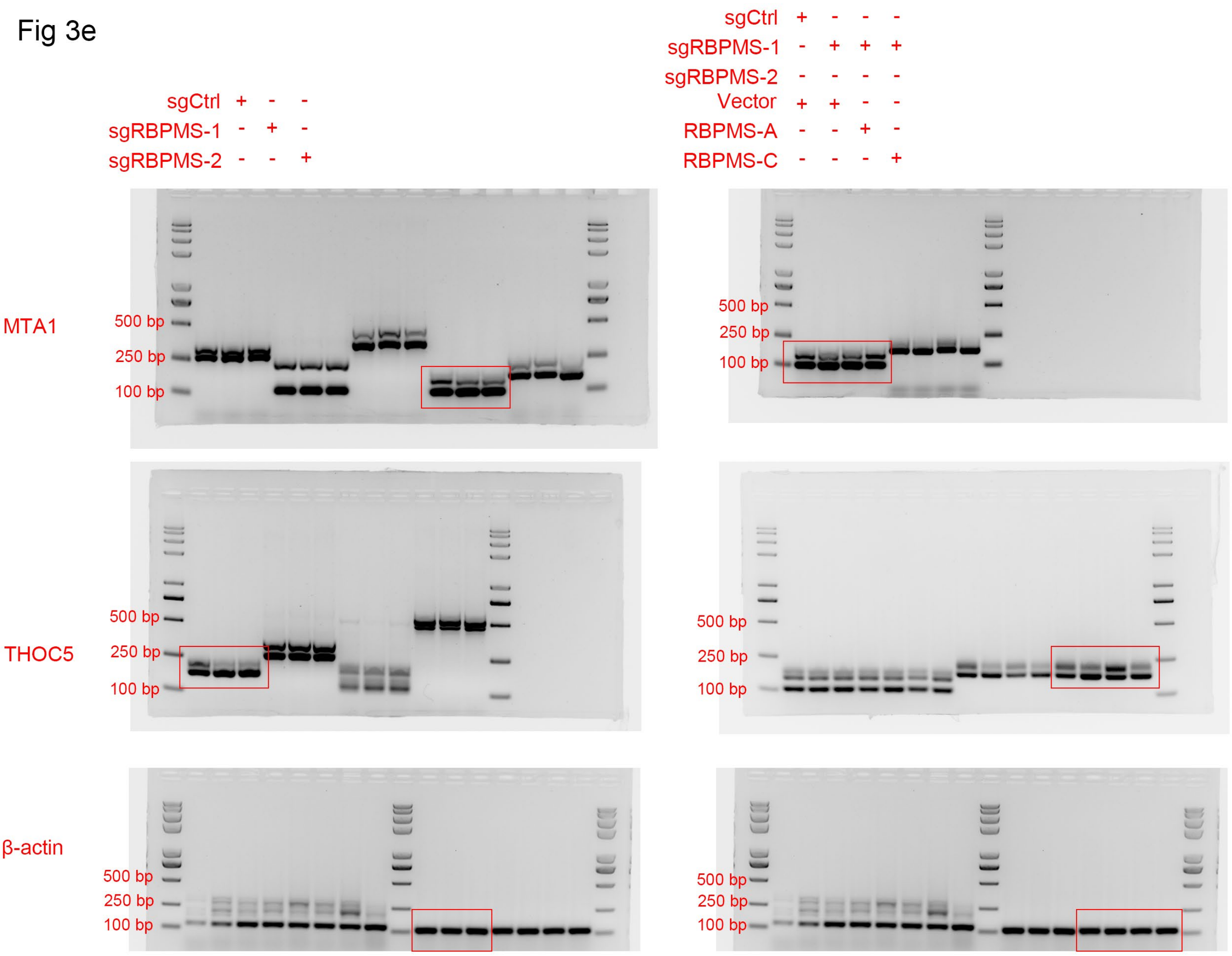

Fig S4i

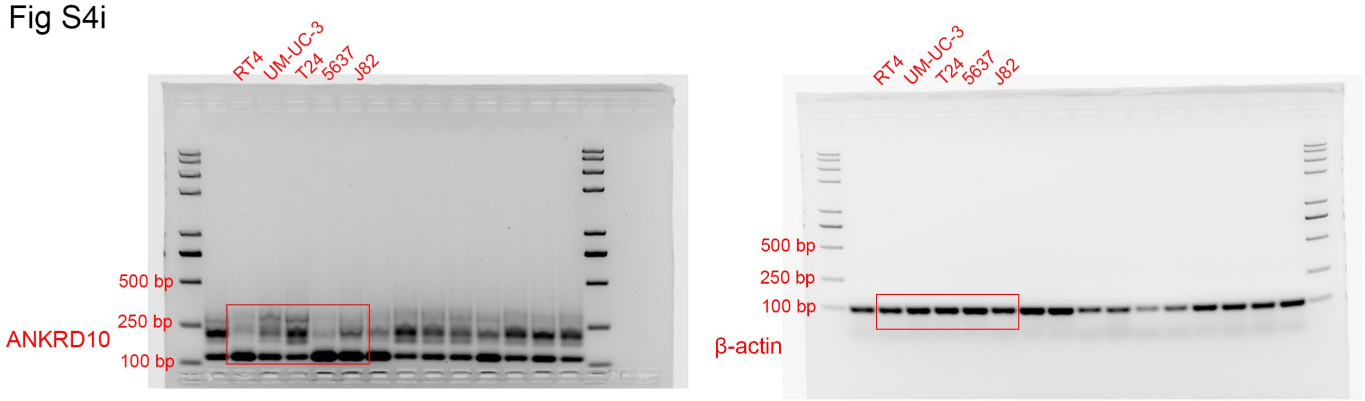

Supplementary Figure 7. Original uncropped agarose gels.

Supplementary Tables 1-6

Supplementary Table 1.The sgRNA or siRNA sequences used in this study.

Gene	No.	siRNA sequence (5'-3')
RBPMS	sgRNA#1	CACCGGCGGAATTTCAGGATCGAAG
	sgRNA#2	CACCGGAGCGAGGCCAACCTTCAGG
ANKRD10-1	siRNA#1	CGACACAGAAGACGATGCTGACAAA
	siRNA#2	CGTTGATAGGGAGTTTGCTGTTGTA
ANKRD10-2	siRNA#1	GCGGGTGGGAAGAGCACACTTATTT
	siRNA#2	CCACTGCGAAGATGGGACTTAACAA

Supplementary Table 2. Details of antibodies.

Protein	Catalog No.	Source	Dilution or amount
RBPMS	ab152101	Abcam	IP/1 μg WB/1:1000 IHC/1:100
ANKRD10	ab204396	Abcam	WB/1:1000
Flag	F1804	Sigma	IP/1 μg WB/1:1000
HA	TA180128	Origene	IP/1 μg WB/1:1000
E-cadherin	20874-1-AP	Proteintech	WB/1:5000
N-cadherin	22018-1-AP	Proteintech	WB/1:2000
MYC	18583	Cell Signaling Technology	WB/1:1000
MYC	ab32072	Abcam	ChIP/8 μg
Vimentin	5741S	Cell Signaling Technology	WB/1:1000
β-actin	sc-47778	Santa Cruz	WB/1:1000
Ki67	ab16667	Abcam	IHC/1:200

WB: Western blot.

IP: immunoprecipitation.

IHC: Immunohistochemistry.

Supplementary Table 3. Primers used in qRT-PCR assays.

Gene	Forward Primer (5'>3')	Reverse Primer (5'>3')
RBPMS-1/4	TTCACTGCATGCCCAGATGC	TTCAGCAGAACTGACGGGAC
RBPMS-2	CCCAGCTCTGTGAAGGTCAG	GCACTATCAGGAGACGGAGC
RBPMS-3	ACACACCTGTCTTTTGTCCACT	TGCTGGTCTGCAGTAGGTTG
RBPMS	AAACAGCCTGTAGGTTTTGTCA	GGAATTTCAGGATCGAAGCGG
ANKRD10	TAGTGCAGTTGGTGAGAGCG	CAGACCAGGCACTGAGGATG
GAPDH	GACTCATGACCACAGTCCATGC	AGAGGCAGGGATGATGTTCTG
MYC	GTCAAGAGGCGAACACACAAC	TTGGACGGACAGGATGTATGC
C1QBP	CACACCGACGGAGACAAAG	GGGAGGGTTTTATGCTTCTGAAT
DDX21	CTTTGCCATCCCTTTGATTGAGA	GTAGGTGCAAGAACCAGTACC
HK2	GAGCCACCACTCACCCTACT	CCAGGCATTCGGCAATGTG
PGK1	TGGACGTTAAAGGGAAGCGG	GCTCATAAGGACTACCGACTTGG
PRMT3	GTACCCTTCTCATACCCCAATGG	GACGAGCAGGTTCTGACATCT
RSL1D1	AAGGCAGTGGACGCTCTCT	AGGAGTTGAATTGGGTTCATCC
WDR43	CCTACTTCGCTTTGGCCTCTA	GAAGGCACGTACTCCTGGTG

Supplementary Table 4. Primers used in RT-PCR assays.

Gene	ASEs	Schematic diagram	Primer	primer sequence	Length of PCR products (bp) IJC/SJC
ANKRD10	SE	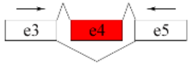	ANKRD10-e3-F<br>ANKRD10-e5-R	GCTCTGGGAGCCTAGAATGC<br>GGCACACTCTTGGAAACCCT	216/122
FAM126A	SE	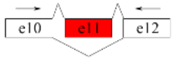	FAM126A-e10-F<br>FAM126A-e12-R	GCTTCATTGCCTCATGGTCC<br>TGATAAACCCGACTGGCTGG	461/246
FN1	SE	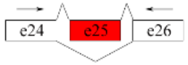	FN1-e24-F<br>FN1-e26-R	TGGTCCATGCTGATCAGAGC<br>TGGTGAATCGCAGGTCAGTG	432/159
CD46	SE	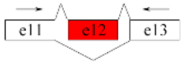	CD46-e11-F<br>CD46-e13-R	TGCCATAGTTGTTGGAGTTGC<br>CTGGCAAACCAGGTTGTGGA	264/171
ZNF195	SE	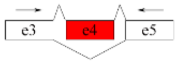	ZNF195-e3-F<br>ZNF195-e5-R	TGCCTGGAGCAACGAAAAGA<br>CTCTGGCAGAAGGTCTTGGG	249/102
MTA1	SE	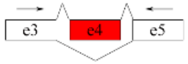	MTA1-e3-F<br>MTA1-e5-R	CATCTCCAGCACCCTCATCG<br>TAGTTTCTCGGGCAGGTCCA	148/97
THOC5	SE	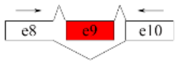	THOC5-e8-F<br>THOC5-e10-R	CGGTGCAGGAGTACCTGTTT<br>AGAGCCTTGGCTTCATCCAC	208/175

Supplementary Table 5. Primers used in ChIP and CLIP assays.

Amplicons	Forward Primer (5'>3')	Reverse Primer (5'>3')
C1QBP	CTGGTCCTCAGTGCGTGACC	GGCAACTGTGTATGTGGGAGTCAC
DDX21	GGCGCCTGTAATCTCAGCAAC	CTGTCGTCCAGGCTGGAGTAC
HK2	GGGTCACCCCGCAGGTAGTC	GCCACGATTCTCTCCACGTG
PGK1	TTGCTGGCCTCCAATCTCA	ATGCGCAGAGTAAGAGACCCAA
PRMT3	GAGAAGACATTCCCAGTGTCAGTCAC	GGCCTCAACATATCCTCGGCC
RSL1D1	GCCAGGAGTTCGAGACCAAC	GATCCTCCGGCTTCAGCTTC
WDR43	CTCTATCTCTCCCTTGGCCACTTG	GGAAGCTAGGTTGGAGGCTCG
ACTIN	GACTTCTAAGTGGCCGCAAG	TTGCCGACTTCAGAGCAAC
CLIP-2	CGCGGCGCTAGTCTCGTTC	-
CLIP-4	GAGGAGCTGCTCTCGCTCC	-
CLIP-6	TAGTGCAGTTGGTGAGAGCG	-
CLIP-8	AAGCAGGAGCCAACATTAACAA	-
CLIP-12	AGTGTGGGAACAAATCGAAAGAG	-
CLIP-15	CAGTAGCTCCGTATCGAATACATTG	-
CLIP-22	GCTCAGAATACCCATGTAGCAGC	-
CLIP-26	GGTGGTAAATCAGGTGCCTGG	-
CLIP-30	CACTGCCTTGGGCTTCATGG	-
CLIP-36	GGTGCAGATAAAATGTCAAATGAAC	-

Supplementary Table 6. Top 250 upregulated genes and Top 250 downregulated genes in Bladder urothelial carcinoma (TCGA-BLCA).

Gene	Mean Tumor TPM/Mean Normal TPM	*p*-value	Expression in tumors compared to normal tissues
MMP11	44.26776982	1.11E-16	up-regulated
CDKN2A	19.54418847	1.62E-12	up-regulated
CST6	17.47117039	1.11E-07	up-regulated
IGF2	9.384093369	6.86E-13	up-regulated
TROAP	7.776817077	0	up-regulated
CENPA	7.646331517	0	up-regulated
LAMC2	7.295745804	4.77E-08	up-regulated
RAC3	7.224348612	1.62E-12	up-regulated
CDH3	7.156214862	1.62E-12	up-regulated
MMP1	7.047038016	2.58E-08	up-regulated
CXCL10	6.983949902	3.91E-09	up-regulated
ETV4	6.845785279	0	up-regulated
NUF2	6.834144763	0	up-regulated
PYCR1	6.728735394	1.62E-12	up-regulated
MYBL2	6.646466139	0	up-regulated
UBE2C	6.591629035	1.62E-12	up-regulated
C9orf140	6.438415151	0	up-regulated
C1QTNF6	6.201936607	1.62E-12	up-regulated
HJURP	6.143465549	1.11E-16	up-regulated
IFI6	6.135108253	1.62E-12	up-regulated
PLA2G2F	6.129250905	1.98E-14	up-regulated
CDC20	6.114245208	1.62E-12	up-regulated
GTSE1	6.103071153	1.62E-12	up-regulated
MELK	6.071222972	1.62E-12	up-regulated

HIST3H2A	6.041338446	0	up-regulated
COL7A1	6.01909207	0	up-regulated
UHRF1	5.92699775	0	up-regulated
PABPC1L	5.909309082	1.62E-12	up-regulated
TPX2	5.896107072	2.56E-14	up-regulated
KIF2C	5.872178649	0	up-regulated
HIST2H2AA3	5.872072223	0	up-regulated
MEST	5.857240961	1.11E-16	up-regulated
C4orf48	5.827795467	1.62E-12	up-regulated
APOC1	5.726332918	0	up-regulated
HIST1H1C	5.688217958	0	up-regulated
CDK1	5.677003336	1.63E-12	up-regulated
PLK1	5.631722068	1.62E-12	up-regulated
HIST1H2BD	5.619375255	1.62E-12	up-regulated
AURKA	5.598180987	1.62E-12	up-regulated
TMEM132A	5.5854427	0	up-regulated
C15orf48	5.537852348	5.45E-12	up-regulated
ISG15	5.530064789	0	up-regulated
CDCA8	5.451747776	1.11E-13	up-regulated
NEK2	5.450991854	7.07E-12	up-regulated
ZWINT	5.404976275	1.48E-14	up-regulated
RECQL4	5.382973496	0	up-regulated
AURKB	5.335282325	1.62E-12	up-regulated
CDC45	5.317990313	1.62E-12	up-regulated
RARRES1	5.304024603	1.79E-08	up-regulated
CCNE1	5.266937104	0	up-regulated

TUBB3	5.153589969	1.29E-13	up-regulated
CDT1	5.108170654	1.62E-12	up-regulated
PRSS8	5.094404474	1.63E-12	up-regulated
CDCA3	4.999527822	2.31E-12	up-regulated
IGSF9	4.942296219	0	up-regulated
FOXM1	4.900550359	4.62E-09	up-regulated
TRIP13	4.888164651	1.63E-12	up-regulated
SPAG5	4.84430554	1.66E-12	up-regulated
ASF1B	4.815257349	2.44E-15	up-regulated
PLAU	4.772188574	0	up-regulated
CCNB2	4.771138417	2.42E-12	up-regulated
CDC6	4.719061826	1.62E-12	up-regulated
ORC6L	4.714315261	4.38E-09	up-regulated
PAQR4	4.671400252	1.62E-12	up-regulated
DLGAP5	4.666207006	4.57E-10	up-regulated
KIFC1	4.633303743	1.48E-11	up-regulated
TK1	4.518413593	3.39E-13	up-regulated
TOP2A	4.514953205	1.51E-08	up-regulated
BIRC5	4.512144071	5.35E-11	up-regulated
PODXL2	4.502193017	0	up-regulated
IER5L	4.499692889	1.62E-12	up-regulated
IFI27	4.489468467	1.62E-12	up-regulated
KIF20A	4.432271596	1.20E-07	up-regulated
GINS1	4.397808272	1.70E-11	up-regulated
RHPN1	4.339830522	0	up-regulated
C19orf46	4.308412464	1.74E-12	up-regulated

CCNB1	4.301390987	4.52E-12	up-regulated
MKI67	4.301276456	2.85E-08	up-regulated
FER1L4	4.270458439	1.85E-10	up-regulated
KIAA0101	4.254729855	1.95E-11	up-regulated
HMGB3	4.248481342	1.62E-12	up-regulated
NDC80	4.243588271	6.83E-10	up-regulated
ARHGAP8	4.218009683	1.11E-16	up-regulated
UBD	4.211815288	3.38E-09	up-regulated
CDCA5	4.199583941	1.53E-09	up-regulated
NUSAP1	4.175164325	6.13E-09	up-regulated
PKMYT1	4.160252266	1.62E-12	up-regulated
HIST1H2BK	4.158773738	0	up-regulated
APOBEC3B	4.140180828	5.40E-12	up-regulated
MCM2	4.078435818	0	up-regulated
SCD	4.056418497	4.16E-11	up-regulated
SLC39A4	4.052828043	0	up-regulated
TACC3	4.031333121	0	up-regulated
E2F1	3.996513356	1.16E-10	up-regulated
CCNA2	3.971075945	1.75E-08	up-regulated
POSTN	3.945140079	2.41E-12	up-regulated
FABP6	3.941355527	3.76E-09	up-regulated
KIF23	3.938269215	6.40E-07	up-regulated
SLC19A1	3.936733372	1.62E-12	up-regulated
CDCA7	3.92935055	1.62E-12	up-regulated
NFKBIL2	3.924966001	1.62E-12	up-regulated
UCA1	3.91233363	1.20E-07	up-regulated

FCGR3A	3.898263173	3.03E-10	up-regulated
GALNT14	3.868988673	1.63E-12	up-regulated
EZH2	3.836395916	1.68E-12	up-regulated
UBE2T	3.825788522	1.57E-10	up-regulated
HIST2H4A	3.822682662	1.62E-12	up-regulated
MAD2L1	3.80569291	2.30E-13	up-regulated
SLC5A6	3.779465435	1.62E-12	up-regulated
MFAP2	3.773338516	0	up-regulated
UPK2	3.772833344	9.53E-07	up-regulated
CEP55	3.734415738	2.46E-06	up-regulated
NUP210	3.716256399	0	up-regulated
HIST2H2BE	3.711377261	1.62E-12	up-regulated
EPR1	3.685057841	1.19E-06	up-regulated
CHTF18	3.676062416	0	up-regulated
BUB1	3.646574079	4.33E-06	up-regulated
CKS2	3.642774282	1.63E-12	up-regulated
ECT2	3.637255913	8.16E-11	up-regulated
C12orf75	3.631420575	1.63E-12	up-regulated
USP18	3.628071747	1.62E-12	up-regulated
KIF11	3.623274869	1.63E-08	up-regulated
STMN1	3.623124718	2.61E-12	up-regulated
CCL18	3.617213342	2.58E-06	up-regulated
C7orf68	3.616521042	3.60E-10	up-regulated
DBNDD1	3.610383708	1.13E-11	up-regulated
PI4KAP1	3.603598506	0	up-regulated
PBK	3.602786661	2.45E-07	up-regulated

TRIB3	3.597568905	1.62E-12	up-regulated
SPAG4	3.586643994	0	up-regulated
FCHO1	3.560860588	4.45E-10	up-regulated
KIFC2	3.549909121	1.62E-12	up-regulated
UNC13D	3.531687369	1.62E-12	up-regulated
TYMP	3.530807084	8.08E-14	up-regulated
ECE2	3.513266571	1.62E-12	up-regulated
VGLL1	3.493412229	6.54E-08	up-regulated
TINAGL1	3.492008642	5.22E-12	up-regulated
CENPF	3.46780223	6.10E-05	up-regulated
RRM2	3.443358866	3.52E-06	up-regulated
GRB7	3.423431282	2.93E-08	up-regulated
IFIT3	3.40993497	2.32E-12	up-regulated
GINS2	3.398942378	1.63E-12	up-regulated
MLF1IP	3.390852816	2.67E-08	up-regulated
LMNB1	3.374652769	6.01E-11	up-regulated
FBXL6	3.371264036	0	up-regulated
TRPM2	3.362171966	1.11E-16	up-regulated
EPCAM	3.359973756	2.52E-10	up-regulated
CHEK1	3.33304175	2.44E-12	up-regulated
RCC1	3.314689078	1.63E-12	up-regulated
PAFAH1B3	3.309582992	1.58E-14	up-regulated
FANCI	3.299327555	9.56E-09	up-regulated
PRC1	3.270936787	4.57E-06	up-regulated
SOX4	3.248579195	8.95E-09	up-regulated
GGH	3.248060247	0.00047981	up-regulated

NFE2L3	3.241204512	1.58E-10	up-regulated
MUC1	3.231469583	4.74E-08	up-regulated
EPSTI1	3.222242456	3.61E-10	up-regulated
SKP2	3.204644254	0	up-regulated
TYMS	3.192681073	6.00E-07	up-regulated
SQLE	3.189482728	4.68E-11	up-regulated
DONSON	3.179291658	0	up-regulated
EIF4EBP1	3.165376797	1.62E-12	up-regulated
C16orf75	3.159303809	2.55E-13	up-regulated
H2AFX	3.153805133	0	up-regulated
CDCA4	3.151062676	1.62E-12	up-regulated
YDJC	3.150965263	1.62E-12	up-regulated
COL17A1	3.14601501	2.60E-07	up-regulated
LY6K	3.145349856	5.36E-10	up-regulated
FASN	3.143046337	1.62E-12	up-regulated
BRCA1	3.131725678	1.64E-12	up-regulated
CENPM	3.130689575	9.20E-14	up-regulated
KRT7	3.129617206	1.33E-08	up-regulated
SAC3D1	3.113574284	1.62E-12	up-regulated
DHCR7	3.110760954	2.52E-12	up-regulated
KPNA2	3.109580368	6.71E-11	up-regulated
GSTM2	3.09985294	1.75E-08	up-regulated
KRT23	3.090809755	3.10E-06	up-regulated
FEN1	3.084675726	4.06E-11	up-regulated
RTKN	3.080821354	1.62E-12	up-regulated
TMEM206	3.079844966	2.24E-12	up-regulated

ADAM8	3.069736862	3.84E-10	up-regulated
PTTG1	3.062154625	6.15E-11	up-regulated
AGRN	3.059789288	4.34E-11	up-regulated
MDK	3.048379051	1.50E-10	up-regulated
TCF19	3.041840581	1.79E-11	up-regulated
TIMELESS	3.04108808	4.65E-13	up-regulated
MCM4	3.040096861	1.62E-12	up-regulated
KRT18	3.039834891	8.33E-11	up-regulated
TTYH3	3.021423606	2.18E-13	up-regulated
TMEM45A	3.015946875	1.44E-08	up-regulated
ANO1	3.012736562	1.77E-09	up-regulated
IRF5	3.007319916	6.25E-12	up-regulated
NCAPG2	3.001080797	3.46E-09	up-regulated
ITPR3	2.991453933	3.33E-16	up-regulated
CKS1B	2.96027082	1.62E-12	up-regulated
GYLTL1B	2.958672931	3.56E-08	up-regulated
CENPH	2.954052521	7.72E-13	up-regulated
ATAD2	2.950377994	2.67E-10	up-regulated
RNASEH2A	2.939659307	1.30E-14	up-regulated
CDKN3	2.934520241	2.57E-06	up-regulated
RFC4	2.933252368	1.62E-12	up-regulated
POC1A	2.933242474	6.81E-10	up-regulated
MTHFD1L	2.921184544	0	up-regulated
CTHRC1	2.913248322	1.27E-10	up-regulated
WDR90	2.90981855	0	up-regulated
NMB	2.907774719	2.18E-12	up-regulated

TLCD1	2.899207996	1.28E-10	up-regulated
C19orf48	2.895094611	1.62E-12	up-regulated
POLD1	2.892967821	1.62E-12	up-regulated
LY6E	2.889856651	3.40E-11	up-regulated
SPHK1	2.877782645	3.38E-12	up-regulated
UBE2S	2.877701264	0	up-regulated
HN1	2.875939047	1.83E-11	up-regulated
MYO19	2.864166546	1.65E-12	up-regulated
WARS	2.858773849	3.75E-11	up-regulated
TFAP2A	2.853540132	2.01E-07	up-regulated
CCNF	2.853077082	7.17E-10	up-regulated
BPGM	2.848976674	1.24E-12	up-regulated
ERBB2	2.848610364	1.57E-06	up-regulated
MMP7	2.842980724	0.00022372	up-regulated
MFSD3	2.840027548	1.09E-11	up-regulated
SC65	2.829291306	1.65E-12	up-regulated
GPR172A	2.828731869	3.12E-13	up-regulated
NSUN5P2	2.825099485	0	up-regulated
FSCN1	2.82338688	6.79E-11	up-regulated
LRRC45	2.812613522	1.62E-12	up-regulated
EFNA4	2.812047308	2.87E-12	up-regulated
WDR34	2.808301555	0	up-regulated
OAS3	2.801741463	3.73E-11	up-regulated
IGFBP3	2.796581359	5.72E-05	up-regulated
TNNI2	2.792556381	2.24E-05	up-regulated
DHRS13	2.791662055	1.07E-10	up-regulated

MDFI	2.790562831	2.30E-08	up-regulated
PGF	2.787214546	1.74E-12	up-regulated
IFI44	2.786659865	6.20E-09	up-regulated
XRCC3	2.777124414	1.63E-12	up-regulated
PLXNA1	2.772907137	1.62E-12	up-regulated
C8orf30A	2.767596613	1.62E-12	up-regulated
UCK2	2.766002678	1.62E-12	up-regulated
LPCAT1	2.765497919	1.63E-12	up-regulated
MIF	2.759621721	1.62E-12	up-regulated
PPP1R14B	2.750141701	1.63E-12	up-regulated
C15orf23	2.737610145	6.63E-08	up-regulated
PSAT1	2.732343692	1.91E-08	up-regulated
MARCKSL1	2.732088324	1.38E-10	up-regulated
SHMT2	2.729825942	1.62E-12	up-regulated
HIST1H2AC	2.729723085	1.95E-12	up-regulated
PCNA	2.727726331	1.67E-12	up-regulated
GDPD3	2.718759151	0.0001612	up-regulated
BCL2L12	2.718662847	1.62E-12	up-regulated
PI16	0.020772075	1.87E-05	down-regulated
SCARA5	0.02522262	2.31E-05	down-regulated
C2orf40	0.040074727	0.00058095	down-regulated
VIT	0.040782295	0.00091822	down-regulated
OGN	0.042131186	0.00030037	down-regulated
C16orf89	0.043344964	2.31E-06	down-regulated
ADH1B	0.043530569	5.75E-05	down-regulated
CLEC3B	0.04558764	2.02E-05	down-regulated

TNXB	0.058793017	3.79E-05	down-regulated
ITGA8	0.058889412	0.00015956	down-regulated
NEGR1	0.059407009	0.00060458	down-regulated
FAM107A	0.063106518	1.62E-05	down-regulated
C7	0.065986068	0.00026482	down-regulated
F10	0.066293109	4.13E-06	down-regulated
PCOLCE2	0.067896541	0.00015622	down-regulated
FXYD1	0.068795394	4.81E-06	down-regulated
GPR133	0.070551197	0.00076126	down-regulated
SDPR	0.072775167	0.00014053	down-regulated
CFD	0.075246818	1.84E-06	down-regulated
NR4A1	0.078079374	8.06E-05	down-regulated
SGCA	0.079249552	0.00091704	down-regulated
IGSF10	0.081293114	0.0002174	down-regulated
FGL2	0.083068499	0.00045608	down-regulated
DPT	0.085265907	0.00025929	down-regulated
PDK4	0.085971389	0.00010379	down-regulated
ITIH5	0.086275635	0.00010419	down-regulated
CCL14	0.089613671	7.52E-05	down-regulated
GSTM5	0.089815302	3.56E-06	down-regulated
TPPP	0.095158727	1.87E-05	down-regulated
PLAC9	0.096975452	1.43E-06	down-regulated
ADAMTS1	0.097798632	0.00010332	down-regulated
ABI3BP	0.100041139	5.55E-05	down-regulated
RCAN2	0.109603427	0.0001788	down-regulated
ADAM33	0.112866375	4.03E-06	down-regulated

MFAP4	0.114231918	2.04E-05	down-regulated
GEM	0.114659867	0.00061803	down-regulated
CHRDL1	0.114936234	0.00055543	down-regulated
FOXF1	0.11544683	3.54E-05	down-regulated
FOS	0.116750895	3.83E-05	down-regulated
PID1	0.116791483	1.90E-05	down-regulated
LOC100302650	0.117445527	0.00021576	down-regulated
AG2	0.117504357	0.00047698	down-regulated
DUSP1	0.118272302	2.69E-06	down-regulated
SPARCL1	0.119100495	0.00012383	down-regulated
HSPB8	0.119846366	0.00061743	down-regulated
SVEP1	0.120119611	0.00051653	down-regulated
NR4A2	0.123463649	0.00099675	down-regulated
TCEAL2	0.123941942	0.00056395	down-regulated
MRVI1	0.124443211	0.0008978	down-regulated
PLCB4	0.124868507	0.00099949	down-regulated
MRGPRF	0.125205902	0.00015814	down-regulated
SLIT3	0.12523704	5.82E-05	down-regulated
DIXDC1	0.125509394	0.00066362	down-regulated
LOC399959	0.129186823	3.82E-05	down-regulated
PDZRN3	0.130323735	0.00053733	down-regulated
ANK2	0.131992741	7.51E-05	down-regulated
PAMR1	0.133866856	1.51E-05	down-regulated
ZFP36	0.136612994	0.00049793	down-regulated
SMOC2	0.136829007	0.00023781	down-regulated
RGS2	0.140185005	0.00056105	down-regulated

KLF2	0.14150403	3.70E-05	down-regulated
COL14A1	0.142162015	0.00013989	down-regulated
COX7A1	0.143875878	0.00033836	down-regulated
GATA6	0.145567166	0.00011834	down-regulated
LRRN4CL	0.146533487	3.49E-06	down-regulated
JAM3	0.149131195	0.00070294	down-regulated
CCDC69	0.149391005	0.00042433	down-regulated
CSRNP1	0.151458979	0.0003222	down-regulated
RAI2	0.151867954	1.58E-05	down-regulated
PHYHIP	0.1526023	1.53E-05	down-regulated
STON1	0.153362468	0.00073574	down-regulated
EPHA3	0.154663791	8.28E-05	down-regulated
PDE5A	0.155070073	0.00054095	down-regulated
C5orf4	0.155454093	0.00011716	down-regulated
HSD17B6	0.155611284	0.00066301	down-regulated
ACACB	0.15711814	0.00025736	down-regulated
PDE2A	0.158287713	0.00010034	down-regulated
WFDC1	0.160044385	0.00018351	down-regulated
DCN	0.161468779	5.60E-05	down-regulated
CELF2	0.163258705	0.00019516	down-regulated
CADM3	0.16337606	0.00010246	down-regulated
ZCCHC24	0.164803153	0.00058481	down-regulated
TPSD1	0.167372251	0.00015842	down-regulated
LAMA2	0.167532619	0.00015544	down-regulated
JAM2	0.168356472	2.13E-05	down-regulated
TMOD1	0.169397088	0.00043677	down-regulated

TSPAN18	0.170825191	0.00086128	down-regulated
CCDC80	0.171136924	0.00072281	down-regulated
FILIP1L	0.171671144	0.00094472	down-regulated
C10orf72	0.173450438	1.76E-05	down-regulated
TCEAL7	0.174752694	7.44E-15	down-regulated
MUSTN1	0.175958388	0.00062167	down-regulated
CBX7	0.177655133	4.05E-05	down-regulated
TPSAB1	0.178397139	0.00023787	down-regulated
HAAO	0.179846234	1.83E-05	down-regulated
ITPR1	0.181864422	0.00064444	down-regulated
PRICKLE2	0.182933989	0.00030457	down-regulated
GSN	0.183157063	5.80E-05	down-regulated
FBLN5	0.183566169	6.13E-05	down-regulated
LAMC3	0.184550467	0.00043685	down-regulated
AQP1	0.185326686	6.14E-05	down-regulated
LTBP4	0.185505239	2.31E-05	down-regulated
SRPX	0.185764964	1.56E-06	down-regulated
TNFAIP8L3	0.187003962	0.0001046	down-regulated
CLDN5	0.187265378	0.00040459	down-regulated
APOLD1	0.187844788	0.0002566	down-regulated
HSPB2	0.188026489	0.00016498	down-regulated
RASGRP2	0.190038784	0.00027727	down-regulated
FGF7	0.191110305	0.00043906	down-regulated
SPON1	0.192044183	3.92E-05	down-regulated
MYADM	0.19483862	3.50E-05	down-regulated
PPAP2B	0.195156402	4.43E-06	down-regulated

CXCL12	0.196199003	0.00028245	down-regulated
KLF4	0.197313956	0.00010064	down-regulated
PODN	0.200247839	0.0002177	down-regulated
CPA3	0.20124435	0.0006371	down-regulated
SYNE1	0.203135599	2.88E-06	down-regulated
MATN2	0.203147837	1.56E-06	down-regulated
OLFML1	0.204234059	0.00017617	down-regulated
CRYM	0.204750581	2.38E-06	down-regulated
FBLN2	0.207993884	0.00012394	down-regulated
NFIX	0.208270405	0.00046233	down-regulated
OLFM1	0.209738123	4.55E-07	down-regulated
INMT	0.210284151	0.00011868	down-regulated
CYBRD1	0.210336123	8.36E-05	down-regulated
SLC24A3	0.211669927	1.75E-05	down-regulated
TPSB2	0.212990397	0.000121	down-regulated
GPR124	0.216347997	0.00016447	down-regulated
BTG2	0.217288397	0.00043894	down-regulated
CSGALNACT1	0.218683633	1.50E-05	down-regulated
NCALD	0.219188422	0.00065535	down-regulated
BDKRB2	0.219521231	0.0001373	down-regulated
TGFBR3	0.221360964	1.98E-05	down-regulated
GAS7	0.222374171	0.00049718	down-regulated
TP53INP2	0.224530278	0.00019716	down-regulated
RHOB	0.225909045	6.29E-05	down-regulated
C16orf45	0.227245926	0.00010106	down-regulated
THSD4	0.227687474	0.00017691	down-regulated

FGFR1	0.228930986	7.12E-08	down-regulated
PER2	0.23007288	0.00023981	down-regulated
BIN1	0.230213114	0.00045504	down-regulated
COLEC12	0.231412891	0.00023917	down-regulated
RNASE4	0.232504484	2.01E-07	down-regulated
GYPC	0.232851938	7.60E-05	down-regulated
BOC	0.233567361	0.00089724	down-regulated
CD34	0.236186031	6.01E-05	down-regulated
DENND2A	0.241549769	0.00012231	down-regulated
ITM2A	0.242603059	0.00032417	down-regulated
PDE4B	0.242988597	0.00023712	down-regulated
DCHS1	0.243345625	0.00099615	down-regulated
TSHZ3	0.243510885	0.00082405	down-regulated
ARHGAP6	0.246874908	1.64E-09	down-regulated
PKIG	0.246891721	0.00098146	down-regulated
FAM149A	0.247455392	3.90E-05	down-regulated
SLC9A9	0.249988487	0	down-regulated
CNRIP1	0.250248906	2.47E-05	down-regulated
PCDH18	0.251764133	0.00031927	down-regulated
STARD13	0.252263457	8.06E-05	down-regulated
EDNRA	0.252995064	0.00026281	down-regulated
FZD7	0.253076553	0.00016136	down-regulated
TENC1	0.255676679	1.58E-05	down-regulated
RHOJ	0.256246228	7.71E-05	down-regulated
SPRY1	0.256651351	3.78E-05	down-regulated
NFIA	0.256754057	9.73E-05	down-regulated

PER1	0.261022587	0	down-regulated
SSPN	0.261553883	0.00037755	down-regulated
PTPRN2	0.261639949	0.00079972	down-regulated
PLSCR4	0.261734517	0.00072499	down-regulated
APBB1	0.261957551	0.00040146	down-regulated
TIPARP	0.262155513	0.00089619	down-regulated
MEF2C	0.26391008	0.0001223	down-regulated
MGLL	0.264325501	0	down-regulated
NPR1	0.268661027	8.69E-09	down-regulated
GRASP	0.27156415	5.61E-05	down-regulated
PRKAR2B	0.271734243	4.66E-15	down-regulated
PMP22	0.276921523	8.24E-05	down-regulated
ZEB2	0.27957198	4.37E-05	down-regulated
MGP	0.281311343	1.63E-06	down-regulated
OLFML3	0.281597369	3.82E-09	down-regulated
THRA	0.282345632	0.00014444	down-regulated
PJA2	0.282513938	0.0002429	down-regulated
SOD3	0.286167814	0.00010274	down-regulated
NPR2	0.286243337	0.00010357	down-regulated
ARMCX1	0.288298936	0.0004413	down-regulated
GNG11	0.290145403	1.89E-05	down-regulated
COL4A6	0.290253579	0.0009226	down-regulated
NR2F1	0.291579442	0.00029524	down-regulated
ALDH2	0.293447172	0.00020439	down-regulated
CCND2	0.294550359	0.00054008	down-regulated
MMP23B	0.295090269	2.61E-08	down-regulated

CPEB2	0.295435606	4.29E-05	down-regulated
SDC2	0.295953303	0.00054486	down-regulated
CLU	0.299936934	4.87E-08	down-regulated
RECK	0.300362179	0.00020197	down-regulated
EPHX2	0.300531543	0	down-regulated
MEIS2	0.30630914	0.00080368	down-regulated
MEIS1	0.306498102	1.58E-05	down-regulated
SYT11	0.306902217	0.00078357	down-regulated
PARM1	0.308543522	3.09E-08	down-regulated
EMILIN1	0.308609579	0.00076396	down-regulated
IGFBP5	0.310946179	1.16E-07	down-regulated
JUNB	0.312471159	0.0003554	down-regulated
FRY	0.315159071	0.00051681	down-regulated
C21orf63	0.315174563	4.85E-13	down-regulated
DLC1	0.318046556	0.00067604	down-regulated
SRF	0.318711466	7.55E-05	down-regulated
TSC22D1	0.319740444	2.34E-05	down-regulated
NAP1L5	0.321363651	1.62E-05	down-regulated
LDB2	0.322440994	2.37E-05	down-regulated
CD302	0.323456796	1.75E-05	down-regulated
RBPMS	0.325403127	0.00073959	down-regulated
CRY2	0.326781288	1.63E-07	down-regulated
LHFP	0.327884962	0.00016152	down-regulated
KLF6	0.33013257	0.00088297	down-regulated
RPS6KA2	0.332059111	1.54E-06	down-regulated
GPR183	0.332103279	5.55E-10	down-regulated

TSPYL2	0.333907218	2.43E-05	down-regulated
ABL1	0.33653024	0.00098117	down-regulated
NFATC4	0.338265783	0.00021524	down-regulated
TACC2	0.33932264	3.80E-06	down-regulated
CC2D2A	0.34085281	0.00056495	down-regulated
FOXN3	0.341456586	0.00073707	down-regulated
LPAR1	0.343697713	4.44E-06	down-regulated
IL33	0.344208117	0.00032369	down-regulated
SCN1B	0.344616111	0.00086211	down-regulated
PGCP	0.346978075	7.90E-05	down-regulated
KANK1	0.347181654	0.00071564	down-regulated
TCEAL1	0.34843051	0.00030123	down-regulated
ABLIM1	0.349235063	8.02E-05	down-regulated
SGCE	0.349973099	0.00015912	down-regulated
TMEM88	0.350025839	0.00015566	down-regulated
TNFSF12	0.354254446	0.00012356	down-regulated
AHNAK	0.355618251	0.00018035	down-regulated
PER3	0.357195301	4.23E-05	down-regulated
NFIB	0.358974955	0.00031546	down-regulated
RCSD1	0.359490457	1.61E-05	down-regulated
SH3BGRL	0.360636582	0.00030193	down-regulated
ATP8B2	0.361762548	2.23E-05	down-regulated
CYYR1	0.363075057	0.00049649	down-regulated
UTRN	0.365163649	1.79E-05	down-regulated
IL11RA	0.366898307	6.52E-08	down-regulated
PCDH7	0.36800244	9.38E-07	down-regulated

MAP3K8	0.368575337	0.00069866	down-regulated
PBX1	0.369060271	0.00043898	down-regulated
PHYHD1	0.370279747	1.18E-08	down-regulated
KCTD12	0.373105406	0.00089521	down-regulated
EIF4E3	0.374672676	0.00047751	down-regulated
PPP1R15A	0.37472476	0.00075679	down-regulated
CX3CL1	0.378018184	4.46E-05	down-regulated
GGTA1	0.382539823	0.00014338	down-regulated
