# Supplementary material for: RBPMS inhibits bladder cancer metastasis by downregulating MYC pathway through alternative splicing of ANKRD10
Source: Commun Biol. 2025 Mar 5;8:367. doi: 10.1038/s42003-025-07842-1 (PMC11882939; doi:10.1038/s42003-025-07842-1)
Supplement: Supplementary file 3 — Description of Additional Supplementary Files [file 42003_2025_7842_MOESM3_ESM.pdf]

Description of Additional Supplementary Files

File name: Supplementary Data 1 – Alternative splicing events in RNA-seq following RBPMS knockout.
Description: The data are as follows: RNA-Seq analysis of splicing events comparing sgRBPMS-1 versus sgCtrl: A3SS (Sheet 1), A5SS (Sheet 2), MXE (Sheet 3), RI (Sheet 4), and SE (Sheet 5); and comparing sgRBPMS-2 versus sgCtrl: A3SS (Sheet 6), A5SS (Sheet 7), MXE (Sheet 8), RI (Sheet 9), and SE (Sheet 10).

File name: Supplementary Data 2 – Source Data.
Description: The source data behind the graphs in the paper.
