# Supplementary material for: RBPMS inhibits bladder cancer metastasis by downregulating MYC pathway through alternative splicing of ANKRD10
Source: Commun Biol. 2025 Mar 5;8:367. doi: 10.1038/s42003-025-07842-1 (PMC11882939; doi:10.1038/s42003-025-07842-1)
Supplement: Supplementary file 6 — Reporting summary [file 42003_2025_7842_MOESM6_ESM.pdf]

nature portfolio

Corresponding author(s): Hang Zheng, Mengxin Lu

Last updated by author(s): Feb 24, 2025

Reporting Summary

Nature Portfolio wishes to improve the reproducibility of the work that we publish. This form provides structure for consistency and transparency in reporting. For further information on Nature Portfolio policies, see our Editorial Policies and the Editorial Policy Checklist.

Statistics

For all statistical analyses, confirm that the following items are present in the figure legend, table legend, main text, or Methods section.

n/a	Confirmed	
☐	☒	The exact sample size (*n*) for each experimental group/condition, given as a discrete number and unit of measurement
☐	☒	A statement on whether measurements were taken from distinct samples or whether the same sample was measured repeatedly
☐	☒	The statistical test(s) used AND whether they are one- or two-sided<br>*Only common tests should be described solely by name; describe more complex techniques in the Methods section.*
☒	☐	A description of all covariates tested
☐	☒	A description of any assumptions or corrections, such as tests of normality and adjustment for multiple comparisons
☐	☒	A full description of the statistical parameters including central tendency (e.g. means) or other basic estimates (e.g. regression coefficient) AND variation (e.g. standard deviation) or associated estimates of uncertainty (e.g. confidence intervals)
☐	☒	For null hypothesis testing, the test statistic (e.g. *F*, *t*, *r*) with confidence intervals, effect sizes, degrees of freedom and *P* value noted<br>*Give P values as exact values whenever suitable.*
☒	☐	For Bayesian analysis, information on the choice of priors and Markov chain Monte Carlo settings
☒	☐	For hierarchical and complex designs, identification of the appropriate level for tests and full reporting of outcomes
☒	☐	Estimates of effect sizes (e.g. Cohen's *d*, Pearson's *r*), indicating how they were calculated

Our web collection on statistics for biologists contains articles on many of the points above.

Software and code

Policy information about availability of computer code

Data collection	The qRT-PCR data: StepOnePlus™ Real-Time PCR System (Thermo Fisher);<br>The RNA-seq data: DNBSEQ-T7 sequencer (MGI Tech Co., Ltd. China);<br>The WB imaging: chemiluminescence and gel imager (BioSpectrum 515 Imaging System, UVP);<br>The Dual-luciferase Reporter Assay data: Dual-luciferase Reporter Assay System kit (Promega);<br>The animal fluorescence detection experiment: IVIS Lumina XRMS Series III (PerkinElmer).
Data analysis	All statistical analyses of the study were performed by R (version 4.1.3) or GraphPad Prism software (version 9) software;<br>All WB quantitative analysis were performed by Image J software (version 1.53);<br>The Gene expression matrices from RNA-seq were analyzed by genome enrichment analysis (GSEA) and gene ontology (GO) through the R package "clusterProfiler" (version 4.8.1);<br>The NanoPhotometer (Cat. #N60, Implen, Germany) to detect the quality and concentration of the extracted RNA.

For manuscripts utilizing custom algorithms or software that are central to the research but not yet described in published literature, software must be made available to editors and reviewers. We strongly encourage code deposition in a community repository (e.g. GitHub). See the Nature Portfolio guidelines for submitting code & software for further information.

Data

Policy information about availability of data

All manuscripts must include a data availability statement. This statement should provide the following information, where applicable:

- Accession codes, unique identifiers, or web links for publicly available datasets
- A description of any restrictions on data availability
- For clinical datasets or third party data, please ensure that the statement adheres to our policy

The RNA-seq data generated in this study have been deposited in the GEO database under accession code (GSE267762). TCGA-BLCA RNA sequencing data were downloaded from the UCSC Xena database (https://xena.ucsc.edu/). The GSE13507 (PMID: 20059769), GSE32548 (PMID: 22685613), GSE32894 (PMID: 22553347), GSE3167 (PMID: 15173019), GSE48075 (PMID: 32546765), GSE83586 (PMID: 28195647), and GSE120736 (PMID: 31735557) datasets were downloaded from the GEO database (https://www.ncbi.nlm.nih.gov/geo/). The remaining data are available within the article and Supplementary Information.

Research involving human participants, their data, or biological material

Policy information about studies with human participants or human data. See also policy information about sex, gender (identity/presentation), and sexual orientation and race, ethnicity and racism.

Reporting on sex and gender	Information was not collected and is not applicable.
Reporting on race, ethnicity, or other socially relevant groupings	Information is not applicable.
Population characteristics	Information is not applicable.
Recruitment	Information is not applicable.
Ethics oversight	Information is not applicable.

Note that full information on the approval of the study protocol must also be provided in the manuscript.

Field-specific reporting

Please select the one below that is the best fit for your research. If you are not sure, read the appropriate sections before making your selection.

☒ Life sciences ☐ Behavioural & social sciences ☐ Ecological, evolutionary & environmental sciences

For a reference copy of the document with all sections, see nature.com/documents/nr-reporting-summary-flat.pdf

Life sciences study design

All studies must disclose on these points even when the disclosure is negative.

Sample size	Samples size for each experiment is indicated in the figures or corresponding figure legends.
Data exclusions	No samples or animals were excluded from the analyses.
Replication	The experiments were successfully repeated. Clear statements have been put into Methods section and Figure legends.
Randomization	The mice were randomly put into separate/groups cages for experiments.
Blinding	For mice studies, the experiments were performed in a blinded fashion when possible. Downstream analyses of mice samples (IHC and H&E) were performed in a blinded fashion, which means that people performing the assays were not aware of the treatment groups until the data analyses were completed.

Reporting for specific materials, systems and methods

We require information from authors about some types of materials, experimental systems and methods used in many studies. Here, indicate whether each material, system or method listed is relevant to your study. If you are not sure if a list item applies to your research, read the appropriate section before selecting a response.

Materials & experimental systems

n/a	Involved in the study
☐	☒ Antibodies
☐	☒ Eukaryotic cell lines
☒	☐ Palaeontology and archaeology
☐	☒ Animals and other organisms
☒	☐ Clinical data
☒	☐ Dual use research of concern
☒	☐ Plants

Methods

n/a	Involved in the study
☒	☐ ChIP-seq
☒	☐ Flow cytometry
☒	☐ MRI-based neuroimaging

Antibodies

Antibodies used

Target, Catalog No., Supplier, Application/Dilution or amount

For Western blot experiment:
Anti-RBPMS, ab152101, Abcam, IP/1 μg WB/1:1000
Anti-ANKRD10, ab204396, Abcam, WB/1:1000
Anti-Flag, F1804, Sigma, IP/1 μg WB/1:1000
Anti-HA,TA180128, Origene, IP/1 μg WB/1:1000
Anti-E-cadherin, 20874-1-AP, Proteintech, WB/1:5000
Anti-N-cadherin, 22018-1-AP, Proteintech, WB/1:2000
Anti-MYC, 18583, Cell Signaling Technology, WB/1:1000
Anti-Vimentin, 5741S, Cell Signaling Technology ,WB/1:1000
Anti-β-actin, sc-47778, Santa Cruz, WB/1:1000
Anti-MYC, ab32072, Abcam, ChIP/8 μg

For immunohistochemistry staining:
Anti-RBPMS, ab152101, 1:100
Anti-Ki67, ab16667, Abcam, 1:200

Validation

All antibodies were purchased from commercial companies, and validated by the data sheets of themanufacturer or citations listed below.
The following primary antibodies were used for Western blot experiments:
1) Anti-RBPMS, validated with Western blot analysis of extracts from A549 whole cell lysate (https://www.abcam.cn/products/primary-antibodies/rbpms-antibody-ab152101.html#lb);
2) Anti-ANKRD10, validated with Western blot analysis of extracts from RT4 and U-251 MG whole cell lysate (https://www.abcam.cn/products/primary-antibodies/ankrd10-antibody-ab204396.html#lb);
3) Anti-Flag, validated with Western blot analysis of Flag protein in CHO lysis solution (https://www.sigmaaldrich.cn/deepweb/assets/sigmaaldrich/product/documents/144/194/vol6_iss2_antiflag_m2.pdf);
4) Anti-HA, validated with Western blot analysis of extracts from HEK293T cells were transfected with HA tagged LGALS3 cDNA for 48 hrs and lysed (https://cdn.origene.com/datasheet/ta180128.pdf);
5) Anti-E-cadherin, validated with Western blot analysis of extracts from HEK293T cells were transfected with sh-E-cadherin transfected A431 cells (https://www.ptgcn.com/products/E-cadherin-Antibody-20874-1-AP.htm);
6) Anti-N-cadherin, validated with Western blot analysis of extracts from HEK293T cells were transfected with sh-Control and sh-N-cadherin (https://www.ptgcn.com/Products/N-cadherin-Antibody-22018-1-AP.htm);
7) Anti-MYC, validated with Western blot analysis of extracts from SCLC-21H, Raji, KG-1a, HT-29, A20, BaF3 and RBL-2H3 whole cell lysates (https://www.cellsignal.cn/products/primary-antibodies/c-myc-e5q6w-rabbit-mab/18583?site-search-type=Products&N=4294956287&Ntt=c-myc&fromPage=plp);
8) Anti-Vimentin, validated with Western blot analysis of extracts from HeLa, NIH/3T3, C6 and COS-7 whole cell lysates (https://www.cellsignal.cn/products/primary-antibodies/vimentin-d21h3-xp-rabbit-mab/5741?site-searchtype=Products&N=4294956287&Ntt=5741s&fromPage=plp&_requestid=5094179);
9) Anti-β-actin, alidated with Western blot analysis of extracts from HeLa, Jurkat, K-562, and A-431 whole cell lysates (https://www.scbt.com/p/beta-actin-antibody-c4);
10) Anti-MYC, validated by ChIP-seq analysis of chromatin extracted from HeLa and NCCIT cells (https://www.abcam.cn/products/primary-antibodies/c-myc-antibody-y69-chip-grade-ab32072.html).

For immunohistochemistry staining:
1) Anti-RBPMS, validated with immunohistochemical analysis of paraffin-embedded Human lung adenocarcinoma tissue (https://www.abcam.cn/products/primary-antibodies/rbpms-antibody-ab152101.html#lb);
2) Anti-Ki67, validated with immunohistochemical analysis of formalin-fixed, paraffin-embedded human tonsil sections (https://www.abcam.cn/products/primary-antibodies/ki67-antibody-sp6-ab16667.html);

For chromatin immunoprecipitation:
Anti-MYC, validated by ChIP-seq analysis of chromatin extracted from HeLa and NCCIT cells (https://www.abcam.cn/products/primary-antibodies/c-myc-antibody-y69-chip-grade-ab32072.html).

Eukaryotic cell lines

Policy information about cell lines and Sex and Gender in Research

Cell line source(s)	RT4, UM-UC-3, T24, 5637, J82, and HEK 293T cells were kindly provided by Cell Bank of Chinese Academy of Science (Shanghai, China).
Authentication	Authentication was performed by Cell Bank, Chinese Academy of Sciences (Shanghai, China).
Mycoplasma contamination	All cell lines were tested negative for mycoplasma contamination.
Commonly misidentified lines (See ICLAC register)	None.

Animals and other research organisms

Policy information about studies involving animals; ARRIVE guidelines recommended for reporting animal research, and Sex and Gender in Research

Laboratory animals	The 4-week-old male nude mice (BALB/c-nude) were purchased from Hubei Beiente Biotechnology Co., Ltd., China. All animals were kept under specific pathogen free (SPF) and temperature-controlled environment with 12h light/12h dark cycle, and free access to food and water.
Wild animals	No wild animals were used in this study.
Reporting on sex	The mice used in the study were all male, because the incidence of bladder cancer is much higher in men than in women (PMID: 27370177).
Field-collected samples	No field-collected samples were used in this study.
Ethics oversight	All work with mice was approved by and performed under the regulations of the Experimental Animal Welfare and Ethics Committee at Zhongnan Hospital of Wuhan University (ZN2022271).

Note that full information on the approval of the study protocol must also be provided in the manuscript.

Plants

Seed stocks	None.
Novel plant genotypes	None.
Authentication	None.
